# An Approach to the Analysis of Menorrhagia
*Adapted from an essay for the Dr. Alfred Edward Aust Lawrence undergraduate Prize of the University of Bristol, 1966.


**Published:** 1967-01

**Authors:** P. F. Fairbrother


### AN APPROACH TO THE ANALYSIS OF MENORRHAGIA*

BY

P. F. FAIRBROTHER

Efforts to control menorrhagia attack the endometrium. It can be dominated by hormones, made redundant by removal of the ovaries, or removed by hysterectomy. These efforts, though effective, are crude in conception because they *attack* the endometrium, which is only the final common pathway for all the many unknown factors that contribute to menorrhagia. Destruction or domination of the final common pathway will be effective in preventing the operation of these factors, but it is surely better to take arms against the factors themselves.

It is first of all necessary to define menorrhagia. In a sense this is impossible owing to lack of knowledge of the distribution of the volume of menstrual loss in the female population at large. It would be easy if it were known that women could be divided strictly into two groups, those with normal menstruation and those with grossly excessive menstrual loss; and nothing in between. It seems more likely that the volume of menstrual blood loss varies continuously in the population from normal to excessive amounts. If this is the case, then menorrhagia is an arbitrarily defined state arising because one or more of the biological variables controlling the volume of blood-loss has altered to increase this volume. An analysis of the factors controlling the volume of blood lost in menorrhagia must begin with normal menstruation. Since the endometrium is the final common pathway for these factors, clues as to their nature might be found there.

### A MODEL FOR " IDEAL " MENSTRUATION

The mature endometrium is supplied by two sets of arteries, the straight basal and the coiled retractile arteries. The element of menstruation is the upper two-thirds of the cylinder of tissue supplied by a coiled artery. The process of menstruation is heralded by prolonged blanching of the cylinder, followed by hæmorrhage into it and to some extent through it to the surface. The element finally sloughs away, and the coiled artery retracts into the remaining endometrium. The shed endometrium is stuffed full of closely packed red cells. The hæmorrhage into the tissue that this betokens is either an accident of nature, or else it is part of the mechanism of menstruation. Supposing it is part of the mechanism of menstruation, the only role it seems to play is mechanical; it loosens and dissects away the cylinder of endometrium, thereby allowing the coiled artery to retract and causing the bleeding to cease.

In this " ideal " model it is assumed that hæmorrhage occurs into the tissue, and that the coiled artery can only retract after sufficient hæmorrhage has occurred to free tissue from it. Such a mechanism leads to the prediction that the volume of hæmorrhage necessary to remove any element of tissue depends entirely upon the tissue. It is important to try to assess the validity of this ideal model, because such will be the validity of deductions made from it. The only relevant experimental situation was provided by Markee in 1940, when he made auto-transplants of the rhesus monkey's endometrium into the

* Adapted from an essay for the Dr. Alfred Edward Aust Lawrence undergraduate Prize of the University of Bristol, 1966.

anterior chamber of the eye. The transplant became vascularized, and the site and volume of most of the hæmorrhage from the transplant was recorded over many cycles. Seventy-five per cent of the bleeding occurred before sloughing took place. This bleeding mostly took the form of capillary or arteriolar bleeding into the cylinder, and then in roughly equal proportions it either immediately broke the surface or formed a hæmatoma which then broke the surface. Four per cent of this fraction of bleeding was ascribed to diapedesis. Only twenty-five per cent of the total bleeding occurred after the slough had separated. Very nearly all of this came from venules, and only a half of one per cent of the total bleeding was due to arterial bleeding into the space left by the separated cylinder. On the basis of these results it seems reasonable to conclude that the sloughing of the cylinder of tissue is an event which, if it does not cause the artery to stop supplying blood, is not followed by further escape of arterial blood. It is possible therefore for the mechanism of the model to be valid. On the other hand, deductions made from the model will only account for at most three-quarters of the hæmorrhage. This need not invalidate the model if it always accounts for three-quarters. The model represents a situation in which all the hæmorrhage occurs into the tissue and none breaks the surface. It is nonsense to suggest that none should break the surface, and to this extent the model is nonsense. However, it seems likely that the hæmorrhage that breaks the surface will bear a constant proportion to that occurring into the tissue, and so the model is probably valid, for although strictly it accounts for only a proportion of the hæmorrhage, that proportion is fixed. If these conclusions derived from an experimental preparation can be applied to man, there seem to be good grounds for hoping that the "ideal" model of menstruation will be valid. In the final event, predictions from the model will have to be tested against observed human behaviour.

In the ideal model, hæmorrhage occurs into the tissue and mechanically dissects it free. As a result of its structure the tissue must be able to absorb only a certain amount of mechanical energy before it floats away. The rate at which blood can supply this energy is the product of its pressure and its rate of flow. If the pressure is constant, the required amount of energy will have been supplied when a given volume of blood has flowed into the tissue. This volume will depend upon how much energy the tissue can absorb. This is a property of the tissue which might be called its "ease of dissectibility". The more energy it can absorb, the less dissectible it is, and *vice versa.* Since the property of dissectibility refers to a unit of tissue, the actual volume of blood required will depend upon the total mass of the tissue involved. The two properties of ideal endometrium upon which the volume of hæmorrhage depends are its mass and its dissectibility; or expressed algebraically,

$$V = k\frac{m}{d}$$

where V represents the volume of menstrual blood, m the endometrial mass, d its dissectibility, and k is a constant. The hypothesis is now written in a form which makes it amenable to direct testing.

### TESTING THE REQUIREMENTS OF THE MODEL

The menstrual blood volume could be determined by measuring the concentration of alkaline hæmatin in a solution of known volume, containing all a woman's sanitary towels. This would give the mass of hæmoglobin lost, and with a knowledge of the blood hæmoglobin concentration the loss of blood could be calculated. The mass of endometrium shed at menstruation would seem a little harder to measure. It could be determined by measuring the wet weight of all vaginal discharge, and subtracting from this the weight of the computed blood loss. Any vaginal secretion would contribute to the wet weight, and so increase the apparent endometrial mass.

Measurement of the dissectibility of the endometrium presents problems, because the endometrium is quite inaccessible. If measurements are to be made they must be indirect, and the chance of being able to do this depends upon finding an accessible tissue whose dissectibility parallels that of the endomertium, and in which dissectibility can be measured. In basic terms dissectibility is the inverse of a tissue's ability to absorb mechanical energy without becoming disrupted. The best way to measure it is therefore to give a standard dose of mechanical energy and measure the resulting degree of disruption. A suitable dose of mechanical energy in this instance would be the injection of a given volume of liquid at constant pressure in a standard time. The degree of disruption could be measured by observing the extent of spread of the liquid after a further fixed interval. It is probably possible to measure dissectibility; it only remains to find an accessible tissue whose dissectibility parallels that of the endometrium. Skin is accessible, and there are encouraging indications that it may also satisfy the second criterion.

The ground substance of a tissue surely determines its dissectibility to some degree. Ground substance is of a similar nature all over the body, having hyaluronic acid as a major part. Any systemically operating factor which changes the properties of hyaluronic acid must affect all tissues, and skin and endometrium will change their properties in parallel. *In vitro*, hyaluronic acid is depolymerised in the absence of a possibly systemically operating factor, namely œstrogen. Furthermore, if it is accepted that menorrhagia can be caused by an altered ground substance, then two observations relating changed skin ground substance with menorrhagia become significant. In the first place, myxœdema is associated with a grossly abnormal interstitial substance in all tissues, including skin, and with menorrhagia. Secondly, Clementson (1962) observed a decrease in skin capillary resistance to deforming forces associated with increased menstrual loss; furthermore, when the skin capillary resistance increased the menstrual loss declined, and *vice versa.* Both menorrhagia and the decline in capillary resistance could be a manifestation of the same change in ground substance. This interpretation of Clementson's work makes the link between the two observations the ground substance, rather than the fragile capillary. According to the ideal model it does not matter whether the capillaries are fragile or robust; but the nature of the ground substance is critical. There seems to be a case for hoping that the dissectibility of the endometrium is paralleled by that of the skin, and so could be measured in the way suggested.

### DIAGNOSTIC IMPLICATIONS OF THE MODEL

Suppose that the hypothesis had been tested in the way described, and had been shown to account satisfactorily for variations in menstrual loss, it would mean that the suppositions concerning the mechanism of menstruation had a substantial degree of truth in them. More important are the practical implications. The equation above is the equation of ideal menstruation, linking the blood loss with the endometrial mass and its dissectibility. For any woman who complains of menorrhagia, the volume of her blood loss could be determined, along with her shed endometrial mass and her skin dissectibility. From the equation it is possible to predict the menstrual loss from the dissectibility and endometrial mass. If this predicted volume coincides with the measured volume, then the mechanism of menstruation is ideal; in which case any menorrhagia can be ascribed to an abnormality in either, or both, of the measured variables. The endometrium might be increased in mass or decreased in dissectibility; the causes of these changes might lie in opposite directions, but if they are to be discovered it is helpful to know in which direction to look—even though there may not be much to see at the present time.

Suppose that the predicted menstrual loss were less than the observed volume, then it must mean that the mechanism of menstruation is non-ideal. It must be abnormal, because bleeding has occurred in excess of that required to remove the tissue cylinders. This could be due to an inefficient sloughing mechanism which allows the mechanical energy of the blood to be wasted, or to excess bleeding after the slough has gone. Also, if the tissue itself were abnormal (say with tuberculous infection, polypi, or carcinoma) it might be expected that principles derived from normal endometrium would not be applicable.

Present day investigations into the causes of menorrhagia seek to find either an abnormal histological pattern in the endometrium or some gross pelvic pathology. There are two serious limitations to this approach. Its basic aim is to associate menorrhagia with some abnormality in either of these spheres; but it is difficult to establish the significance of any such associations. The incidence of the abnormality must be known in the general population and in menorrhagia, and it is only significantly associated with menorrhagia if the second incidence exceeds the first. Doubt arises because the incidence of the abnormality in the general population is often not known. Secondly, supposing that a significant association has been established, it does not imply a causative link; and even if there was a causative link, the mechanism remains obscure. On the other hand, the endometrial mass and its dissectibility are two properties which can be seen to control, at least in part, the menstrual flow. It is rational to want to know, in the first instance, what these variables are.

There is a web of causation in menorrhagia. It is a fundamental problem of medicine to unravel this. Menorrhagia should be divided into two groups. The first is an " ideal " group, where the menstrual volume is predictable from the ideal equation, and this should be subdivided according to which of the two variables, endometrial mass or dissectibility, is found to be abnormal. The second is a " non-ideal " group, which requires further analysis. This is a rational method of differentiating menorrhagia, and it may perhaps reveal unknown causes of this symptom. The alternative is to eliminate all known associations of menorrhagia. As the associations become more numerous, their elimination becomes more tedious; furthermore, this is not an approach that is likely to lead to a better understanding of the problem. Since these associations probably operate through one, two, both, or neither of the two variables already mentioned, measurement of these variables will be a useful step in differential diagnosis.

REFERENCES

Clementson, C. A. B., Blair, L. M., and Reed, D. H. (1962), *Amer. J. Obst. Gyn.*, **83**, 1269.

Markee, J. E. (1940), Contrib. *Embryol.* No. 177, Vol. 28.

